# Long-Term Effects of Climate Variability on Seed Rain Dynamics of Four Fagaceae Sympatric Species in Qinling Mountains, China

**DOI:** 10.3390/biology11040533

**Published:** 2022-03-30

**Authors:** Jing Wang, Xiang Hou, Bo Zhang, Ning Han, Tuo Feng, Xiaolei An, Xiaoning Chen, Jidong Zhao, Gang Chang

**Affiliations:** 1 Shaanxi Institute of Zoology, Xi’an 710032, China; wangjing122411@yeah.net (J.W.); hx426108@163.com (X.H.); mirrorning@163.com (N.H.); fengtuo@ms.xab.ac.cn (T.F.); 13609166855@163.com (X.A.); chenxiaoning1985@163.com (X.C.); jdzhao18@gmail.com (J.Z.); 2 College of Biology Pharmacy and Food Engineering, Shangluo University, Shangluo 726000, China; bobo198632@snnu.edu.cn

**Keywords:** plant phenology, Fagaceae, sympatric species, seed rain dynamics, climate variability

## Abstract

**Simple Summary:**

The external climate is an important factor affecting plant phenology. In this study, we monitored seed rain of four sympatrically distributed Fagaceae species in the Qinling Mountains of China for 10 consecutive years and also collected local climate data to clarify how the seed rain dynamics of four sympatric species changed, whether and how climate variability will affect seed rain drop dynamics of the four sympatric species, and how can the four species better coexist in the same domain. We found there were differences in the seed rain dynamics among the four species, which indicated that there was no concentrated flowering and fruiting among different species. Thus, they could well avoid fierce competition for similar resources, which was also an important reason why different species could co-exist well in the same domain. In addition, our study also found that the seed rain dynamics of these four plants were different in response to different climatic factors, which may be also conducive to their better sympatric distribution.

**Abstract:**

Seed rain, as the beginning of species dispersal, is a key process for forest structure and regeneration. In this study, the seed rain of four Fagaceae sympatric plant species (*Castanea* *mollissima*, *Quercus aliena*, *Quercus variabilis,* and *Quercus serrata*) in the Qinling Mountains were monitored for ten consecutive years, and the responses of seed rain dynamics of the four species to major climatic factors (temperature and precipitation) were analyzed. We found there were significant differences in the seed rain dynamics between *C. mollissima* of *Castanea* and the other three species of *Quercus* in the initial period and end period and the duration of the whole seed rain process among the 10 years. This could indicate to some extent that there was no concentrated flowering and fruiting among different plants of different genera, and they could well avoid fierce competition for similar resources and coexist in the same region. This may also be a reproductive strategy for plants. Seed rain dynamics of different plant species had different sensitivities to climate factors (temperature and precipitation), which indicated that mainly because of their different responses to climate factors, they could well avoid fierce competition for similar climate resources. In addition, the differences in seed rain dropping dynamics could reduce consumption in large numbers by seed predators, thereby promoting their own dispersal and regeneration. All of the above contribute to their better coexistence in the same domain.

## 1. Introduction

Plant phenology refers to the annual seasonal variation of vegetative growth and reproductive growth of plants affected by biological factors (genetics, etc.) and abiotic factors (climate, etc.), such as the budding, leaf-spreading, flowering, fruiting, and leaf-falling of plants [1,2,3,4]. Studies have shown that climate factors can directly or indirectly affect the phenological characteristics of plants, in which temperature and precipitation are considered to be the most important climate factors affecting plant phenology [4,5,6]. Temperature has a strong influence on plant phenology; for example, the spring phenology of plants (especially flowering) is mainly controlled by early temperature, if the spring temperature is higher, the earlier the plant phenology, and the lower the spring temperature, the later the plant phenology [1,2,7]. Precipitation also has important influences on plant phenology. Precipitation not only has an important influence on flowering phenology of plants but also has a very important influence on fruiting phenology of plants [8]. In general, the response degree of phenology of different plants to climate factors is also different. At the same time, many plant phenologies are not affected by a single climate factor but may be affected by a variety of climate factors [9].

Seed is an important organ for sexual reproduction of plants. When the seed is mature, it spreads from the mother plant to the surrounding, thus forming seed rain. Seed rain refers to the number of seeds dropped from the mother tree in a specific time and space and is a visual description of seeds or propagules spreading around [10,11]. The dynamic change of seed rain affects the predation, dispersal, and caching of animals and also affects the storage and dynamics of soil seed banks, thus affecting the seedling establishment and plant regeneration [12]. Therefore, it is of great significance to study the dynamics of seed rain stage in order to understand the regeneration strategy and maintenance mechanism of plant population and community. Seed rain, as the beginning of species dispersal, is a key process for forest structure and regeneration and determines the demographic potential of populations [13,14,15]. The processes occurring during the early stages of a plant’s life play a major role in population dynamics [16,17] and the maintenance of seed consumers [18]. The spatial distribution of seed rain shows heterogeneity, and seed rain usually has seasonal dynamics and interannual variations. The characteristics of transformation, the biological factors (e.g., plant species, plant height, seed weight, seed dispersal mode, etc.), environmental factors (terrain, slope position, slope direction, etc.), and climate factors (e.g., temperature, precipitation and sunshine length, etc.) can obviously affect the seed rain drop dynamics [19,20]. Seed rains usually have significant seasonal dynamics, the seasonal dynamics of seed rain can be divided into three stages: initial stage, peak stage, and end stage, which have obvious characteristics of single-peak curve. Severe excessive or insufficient sunshine, temperature, and precipitation will affect the development of flowering, fruiting, and young fruit of plants [21]. Plants bloom and produce pollen in large numbers in certain years with favorable weather and use wind pollinators or insects to improve pollination efficiency, which is conducive to fruit bearing. If the precipitation during the flowering period is too high, the pollination activities of insects and other insects may be affected, and the fertilization may be poor or even cause only flowering but no fruit [22]. Thus, climatic factors (temperature and precipitation, light, etc.) may be important reasons for the fluctuations of seed rain drop.

The Fagaceae plant species are widely distributed and dominant trees in the Qinling Mountains, China; they are important components in forest ecosystem and play important roles in the functioning of ecosystems by providing food and habitat for a wide range of animals [23,24,25,26]. Thus, investigations of change in Fagaceae plants masting are very important. However, evidence of the effects of climatic fluctuations on seed rain drops are still very scarce. In this study, we selected four sympatric dominant Fagaceae species, namely *C. mollissima*, *Q. aliena*, *Q. variabilis*, and *Q. serrata,* that are widely distributed and dominant trees in the Qinling Mountains, China. We conducted a 10-year, experimental gathering of data to examine the effects of climatic fluctuations on seed rain dynamics and to test the following scientific questions: (1) How did the seed rain dynamics of four sympatric Fagaceae species change? (2) Will climate variability affect seed rain drop dynamics of the four sympatric Fagaceae species? (3) If so, how does seed rain dynamics respond to climate variability?

## 2. Materials and Methods

### 2.1. Study Site and Species

We carried out this study from 2011 to 2020 in the deciduous broadleaved forests (elevation height below 2000 m) of the Foping National Nature Reserve (33°33′ N~33°46′ N, 107°41′ E~107°55′ E) on the south slopes of the Qinling Mountains in Shaanxi Province, central China. The study site is located at the northern edge of the transition zone between the north subtropical and warm temperate zones. Mean annual temperature was 11.5 °C (mean minimum and maximum temperature are −12.9 °C and 37.0 °C, respectively), and mean annual precipitation was 924 mm [27]. The natural vegetation types are divided into three vertical natural zones by elevation height: conifer forest (above 2500 m), mixed broad-leaved and conifer forest (between 2000 and 2500 m), and deciduous broad-leaved forest (below 2000 m) [28]. Our study site is located in the deciduous broad-leaved forest, and the vegetation is dominated by the Fagaceae species *C. mollissima*, *Q. variabilis*, *Q. aliena,* and *Q. serrata*; the Betulaceae species *Betula luminifera;* and the Pinaceae Lindl species *Pinus tabulaeformis* [25,26,27,28].

In this study, the four sympatric species are dominant species widely distributed in the Qinling Mountains and occupy an important position in the ecosystem of the Qinling Mountains. The four sympatric plant species all belong to Fagaceae. *C. mollissima* belongs to the *Castanea*, is a deciduous tree, blooms from April to June, and has fruit ripening from September to October. The other three kinds, *Q. variabilis*, *Q. aliena,* and *Q. serrata,* all belong to the *Quercus*, and the three plants have similar flowering and fruiting periods, with flowering from March to May and fruiting from September to October.

### 2.2. Seed Rain Dropping Phenology and Climate Data Collection

To investigate seed rain dynamics, we used seed rain collection traps to monitor seed rain drops. We randomly selected eight fruiting trees each of *C. mollissima*, *Q. aliena*, *Q. variabilis,* and *Q. serrata* trees (a total of 32 sample trees) in the study site, and each tree was tagged with an individual number. To collect mature seeds, we set up a seed rain collection trap in the east, west, north, and south directions under the canopy of each tree, respectively (i.e., each tree has four seed rain collection traps). A 1 m^2^ seed rain collection trap was set 1.5 m above the ground under each canopy to catch fallen seeds and other debris and to prevent the seeds from falling into the frame and bouncing off or being eaten by other animals [25,27,28]. Each year, seed rain dropping collections were conducted weekly between 20th August (start of seeds rain) and 24th November (end of seed rain period) during 2011–2020. The seed dropping phenology dates are expressed as the day of year (counted from 1 January of the same year). Seed start dropping was defined as the start of one seed (i.e., seed rain initial period), seed peak dropping was defined as the highest abundance of seeds (i.e., seed rain peak period), and the end of seed dropping was defined as no seeds in the later collection (i.e., seed rain end period).

The monthly record of climatic variables from Foping on the south slopes of the Qinling Mountains in Shaanxi Province, Central China during 2011–2020, including temperature and precipitation, were derived from the China Meteorological Data Service Center (CMDC) (http://data.cma.cn/) (accessed on 1 March 2021), which is publicly accessible. Temperature includes average temperature (T, °C), average maximum temperature (Tmax, °C), and average minimum temperature (Tmin, °C). Precipitation was selected as accumulated precipitation (mm). Because time lags of climate effects on seed dropping phenology, we selected temperature and precipitation from last month (represented by t-1), the month before the last month (represented by t-2), and the month before the last two months (represented by t-3). Spring season and autumn season are also important for plant growth, so we also selected temperature and precipitation in the spring season (March to May) of the current year and autumn season (September to November) of the last year.

### 2.3. Data Analysis

We used the day of year to express the seed dropping phenology dates, which counted from 1 January of the same year. Generalized additive models (GAM) [29] with a quasi-Poisson distribution family (link function was log) were used to analyze the seed dropping phenology. All analyses were carried out in R (version 4.0.3) via the mgcv library [30,31]. The optimal roughness of the smooth terms was determined by minimizing the generalized cross-validation value (GCV) [32]. The GCV of a model is a proxy for the model’s out-of-sample predictive mean squared error [33] and was also used to compare alternative model formulations. As model selection criterion, we used the GCV, deviance explained value, and the ecological theory criterion [34]. A model with lower GCV had more predictive power and was hence preferred. Higher deviance explained value and lower GCV indicated a better fit of the model.

The initial candidate model formula is given by Equation (1)

(1) $$D_{i}=a+b\left(T_{i}\right)+c\left(P_{i}\right)+d\left(T_{i}:P_{i}\right)+\varepsilon_{i}$$

Here, $D_{i}$ is the natural logarithm of the day of year of seed dropping phenology in the year *i.* Parameter a is the overall intercept. $b\left(T_{i}\right)$ is a smooth function of temperature in the last month, the month before the last month, the month before the last two months, spring season of the current year, and autumn season of the last year (cubic regression spline function). $c\left(P_{i}\right)$ is a smooth function of total monthly precipitation in the last month, the month before the last month, the month before the last two months, spring season of the current year, and autumn season of the last year (cubic regression spline function). $d\left(T_{i}:P_{i}\right)$ is a smooth function of interaction effect between temperature and precipitation in the last month, the month before the last month, the month before the last two months, spring season of the current year, and autumn season of the last year on the seed dropping phenology (cubic regression spline function). $\varepsilon_{i}$ is the uncorrelated random errors of zero mean and finite variance.

We used Tukey’s HSD (honestly significant difference) to test the difference among the species of seed dropping times.

## 3. Results

### 3.1. Interannual Dynamics of Seed Rain

Our results showed that seed rain drop of the four sympatric Fagaceae species all had obvious dynamics, with obvious initial period, peak period, and end period. There are some differences in seed rain dynamics among the four species. The seed rain initial period of *C. mollissima* was mainly concentrated in early September, the peak period mainly in late September, and the end period mainly in early November, while the seed rain initial period of the other three species of *Quercus* was mainly concentrated in mid-late August, the peak period mainly in early October, and the end period mainly in mid-late November.

**Figure 1:**
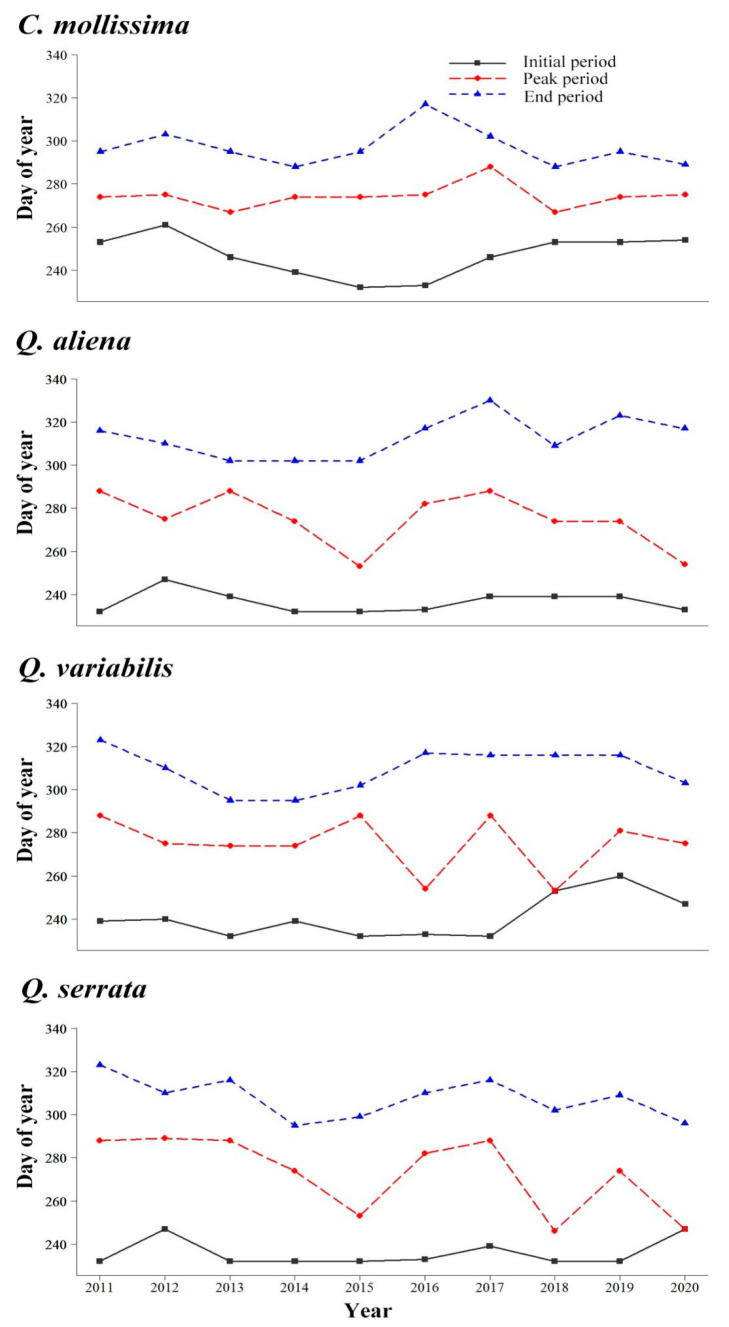
Interannual dynamics of seed rain for the four sympatric plants during 2011–2020.

There were also some differences in seed rain dynamics of the four Fagaceae species during 2011–2020. On the initial period of seed rain, *C. mollissima,* respectively, with *Q. aliena* and *Q. serrata* had significant differences (*p* < 0.05; *p* < 0.05, respectively) but no significant differences with *Q. variabilis* (*p* = 0.30) (Figure 1). On the peak period of seed rain, there were all no significant differences among the four plants (all *p* > 0.05) (Figure 1). At the end period of seed rain, *C. mollissima,* respectively, with *Q. aliena* and *Q. variabilis* had significant differences (*p* < 0.01; *p* < 0.05, respectively) but no significant differences with *Q. serrata* (*p* = 0.06) (Figure 1). For the three species of *Quercus*, there were no significant differences in the initial, peak, and end periods of seed rain among the three species of *Quercus* (all *p* > 0.05).

### 3.2. Climate Variation Drives Seed Rain Dynamics

#### 3.2.1. Response of Seed Rain Dynamics to Temperature Variation

**Figure 2:**
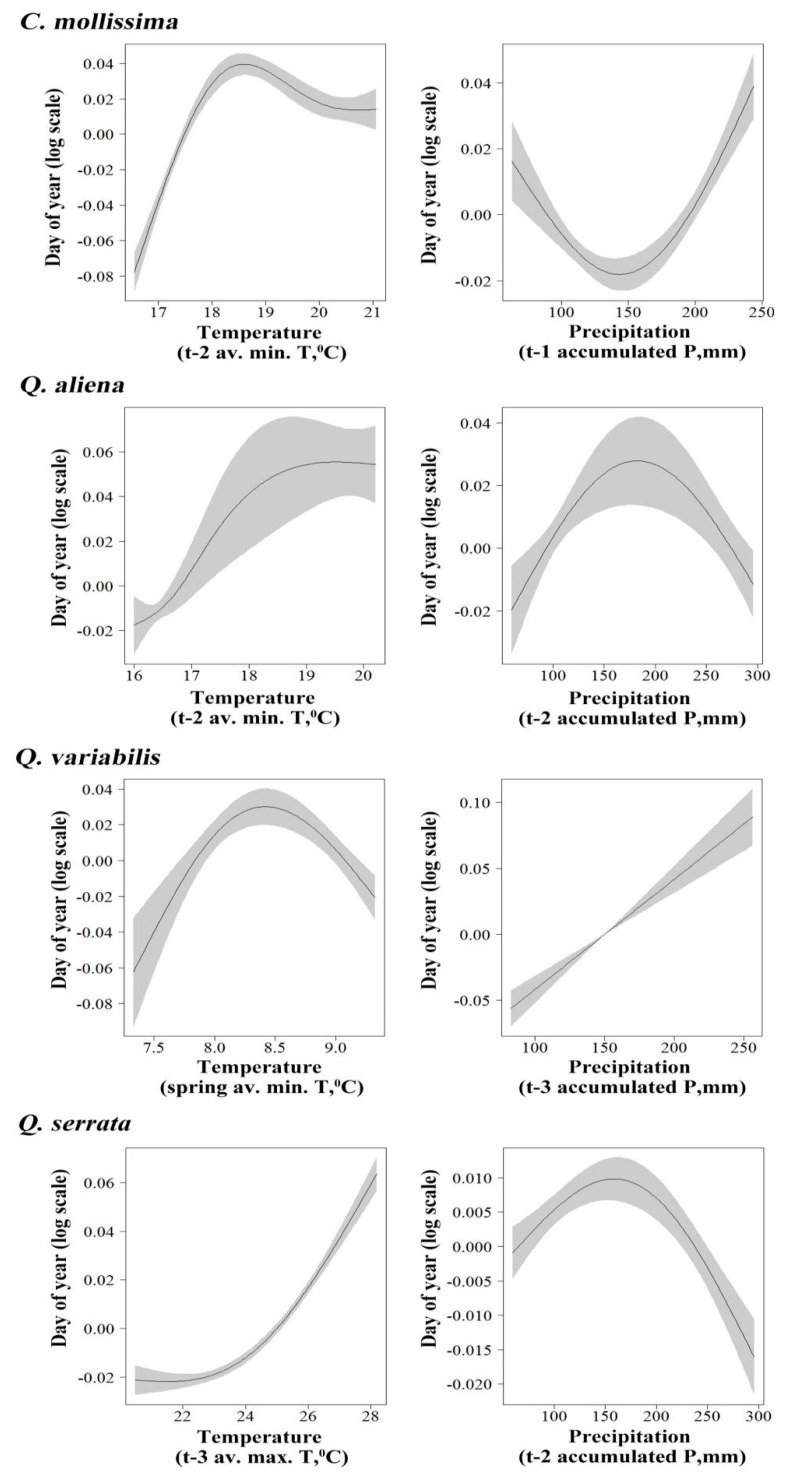
Partial effects from the GAM model on the seed rain initial period (day of year) among four species versus t-2 average minimum temperature and t-1 accumulated precipitation for *C. mollissima* seeds, t-2 average minimum temperature and t-2 accumulated precipitation for *Q. aliena* seeds, spring season average minimum temperature and t-3 accumulated precipitation for *Q. variabilis* seeds, and t-3 average maximum temperature and t-2 accumulated precipitation for *Q. serrata* seeds. Shaded areas are 95% confidence bands.

**Figure 3:**
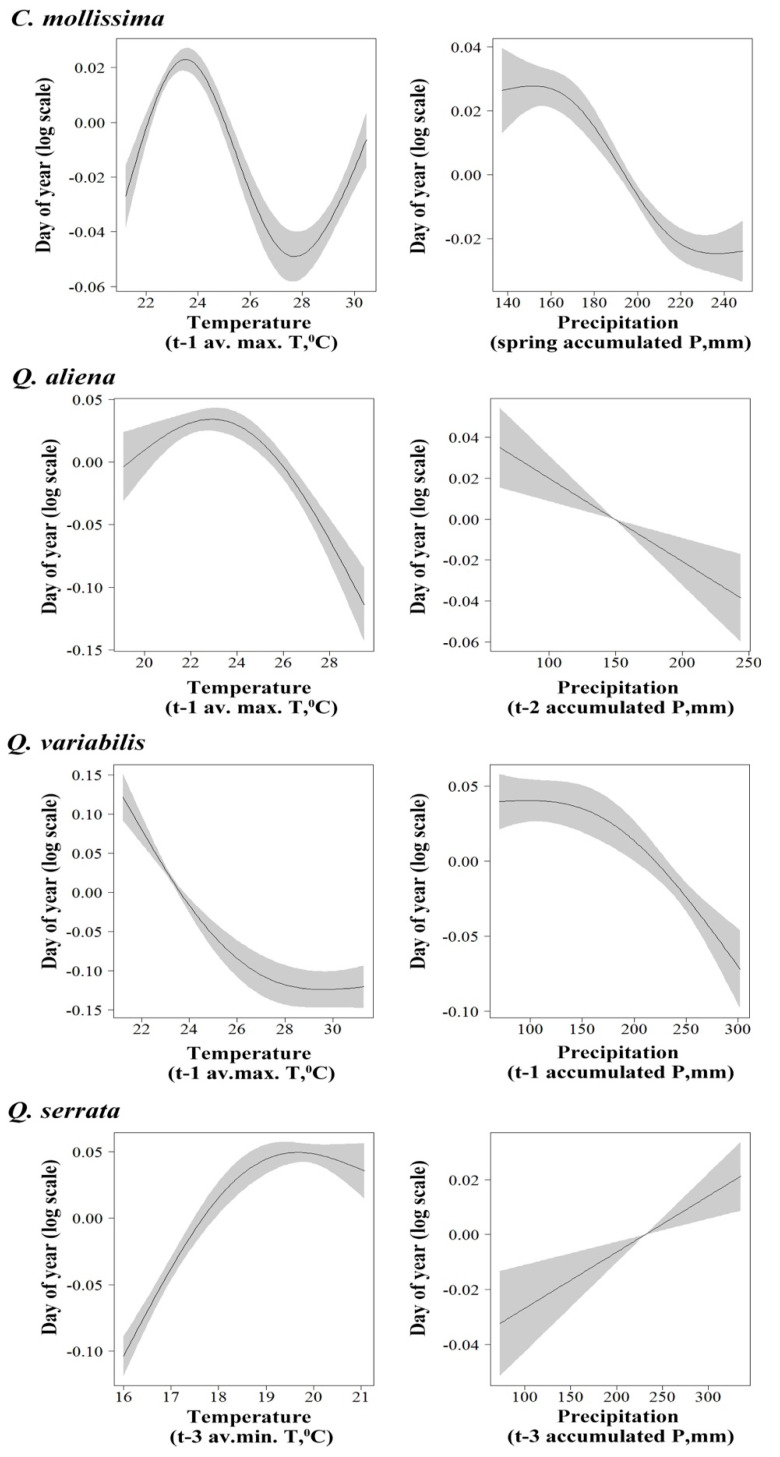
Partial effects from the GAM model on the seed rain peak period (day of year) among four species versus t-1 average maximum temperature and spring season accumulated precipitation for *C. mollissima* seeds, t-1 average maximum temperature and t-2 accumulated precipitation for *Q. aliena* seeds, t-1 average maximum temperature and t-1 accumulated precipitation for *Q. variabilis* seeds, and t-3 average minimum temperature and t-3 accumulated precipitation for *Q. serrata* seeds. Shaded areas are 95% confidence bands.

**Figure 4:**
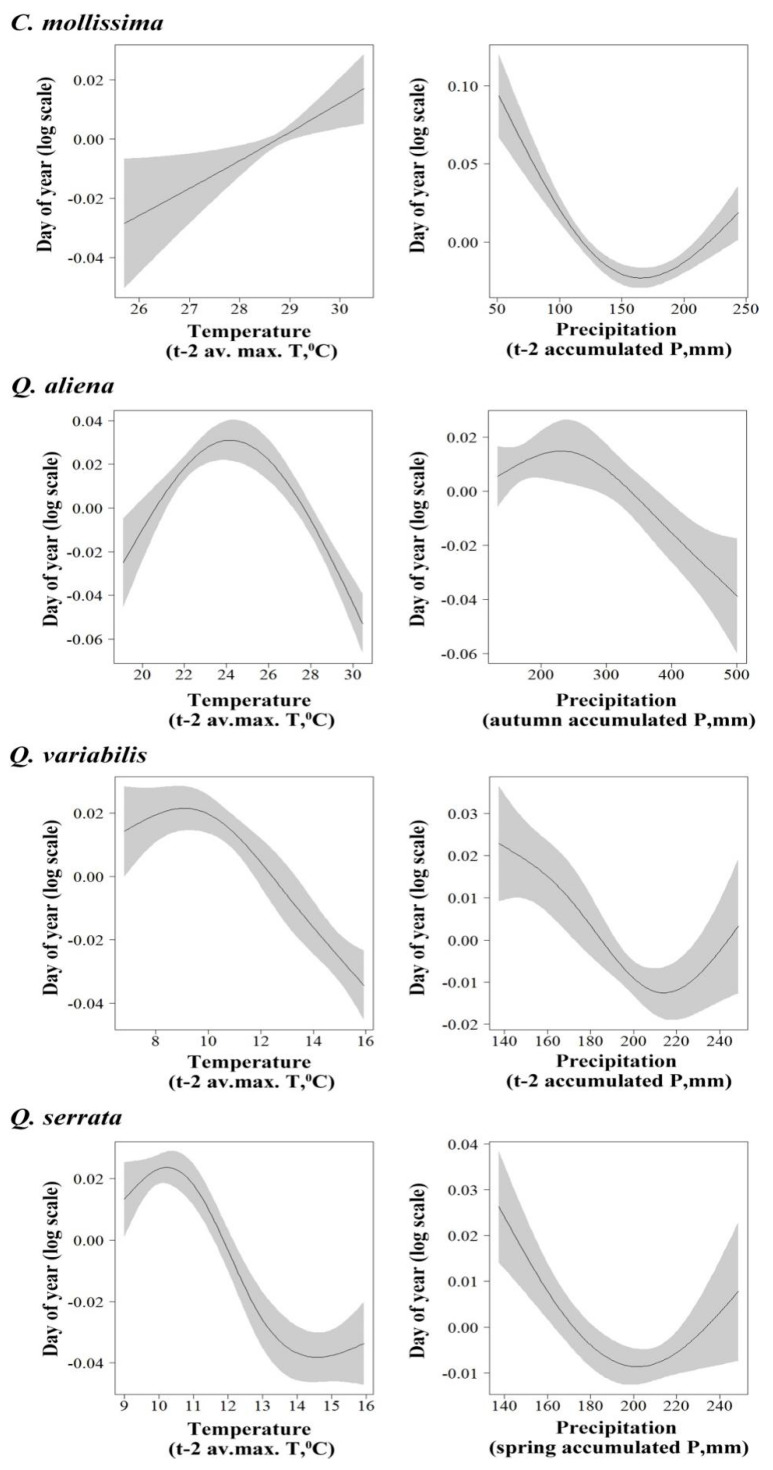
Partial effects from the GAM model on seed rain end period (day of year) among four species versus t-2 average maximum temperature and t-2 accumulated precipitation for *C. mollissima* seeds, t-2 average maximum temperature and autumn season accumulated precipitation for *Q. aliena* seeds, t-2 average maximum temperature and t-2 accumulated precipitation for *Q. variabilis* seeds, and t-2 average maximum temperature and spring season accumulated precipitation for *Q. serrata* seeds. Shaded areas are 95% confidence bands.

By using GAM analysis, we explored the relationship between seed rain dynamics of the four species and temperature variation. For *C. mollissima*, our results showed a nonlinear relationship between seed rain initial period and t-2 average minimum temperature (*F* = 71.31, *p* < 0.01) (Figure 2) and between peak period and t-1 average maximum temperature (*F* = 54.43, *p* < 0.01) (Figure 3) and a linear positive relationship between seed end period and t-2 average maximum temperature (*F* = 6.457, *p* < 0.05) (Figure 4). For *Q. aliena*, a nonlinear relationship was found between initial period and t-2 average minimum temperature (*F* = 21.41, *p* < 0.01) (Figure 2), between peak period and t-1 average maximum temperature (*F* = 30.24, *p* < 0.01) (Figure 3), and between end period and t-2 average maximum temperature (*F* = 31.59, *p* < 0.01) (Figure 4). For *Q. variabilis*, a nonlinear relationship was found between initial period and spring season average minimum temperature (*F* = 17.88, *p* < 0.01) (Figure 2), between end period and t-2 average maximum temperature (*F* = 44.900, *p* < 0.01) (Figure 4), and a nonlinear negative relationship between peak period and t-1 average maximum temperature (*F* = 57.42, *p* < 0.01) (Figure 3). For *Q. serrata*, there was a nonlinear positive relationship between initial period and t-3 average maximum temperature (*F* = 175.6, *p* < 0.01) (Figure 2) and a nonlinear relationship between peak period and t-3 average minimum temperature (*F* = 100.78, *p* < 0.01) (Figure 3) and between end period and t-2 average maximum temperature (*F* = 16.779, *p* < 0.01) (Figure 4).

#### 3.2.2. Response of Seed Rain Dynamics to Precipitation Variation

GAM analysis revealed that a nonlinear correlation between seed rain initial period of *C. mollissima* and t-1 accumulated precipitation (*F* = 33.04, *p* < 0.01) (Figure 2), between peak period and spring season accumulated precipitation (*F* = 29.53, *p* < 0.05) (Figure 3), and also between end period and t-2 accumulated precipitation (*F* = 25.508, *p* < 0.01) (Figure 4). For *Q. aliena*, there was a nonlinear correlation between initial period and t-2 accumulated precipitation (*F* = 8.94, *p* < 0.05) (Figure 2) and between end period and autumn season accumulated precipitation (*F* = 5.88, *p* < 0.05) (Figure 4) and a negative linear relation between peak period and t-2 accumulated precipitation (*F* = 12.85, *p* < 0.05) (Figure 3). For *Q. variabilis*, there was a positive linear relation between initial period and t-3 accumulated precipitation (*F* = 66.73, *p* < 0.01) (Figure 2) and a nonlinear correlation between peak period and t-1 accumulated precipitation (*F* = 16.70, *p* < 0.01) (Figure 3) and between end period and t-2 accumulated precipitation (*F* = 9.398, *p* < 0.05) (Figure 4). For *Q. serrata*, there was a nonlinear correlation between initial period and t-2 accumulated precipitation (*F* = 23.38, *p* < 0.01) (Figure 2) and between end period and spring season accumulated precipitation (*F* = 7.026, *p* < 0.05) (Figure 4) and a positive linear relation between peak period and t-3 accumulated precipitation (*F* = 11.48, *p* < 0.05) (Figure 3).

## 4. Discussion

In this study, we used 10-year consecutive seed rain monitoring data of four Fagaceae sympatric species and climate variables, including temperature and precipitation, to investigate the interannual dynamic changes of seed rain as well as the differences of seed rain dynamics among the four plants and also to verify how climate variability affects the dynamic changes of seed rain.

### 4.1. Interannual Dynamics of Seed Rain

The temporal dynamics of seed rain reflect the change of seed composition during seed rain in different periods; meanwhile, time dynamics is one of the main characteristics of seed rain dropping [35]. In our study, whether *C. mollissima* of *Castanea* or *Q. aliena*, *Q. variabilis,* and *Q. serrata* of *Quercus*, and they all had obvious initial, peak, and end period of seed rain dropping. The seed rain dropping of *C. mollissima* began in early September, peaked mainly from late September to early October, and ended in early November. In the other three species of *Quercus*, the seed rain dropping mainly concentrated from mid-August to mid-late November, and the peak period mainly concentrated from late September to mid-October. The seed rain process of *C. mollissima* mainly lasted about 50 days, while the other three species lasted longer, almost more than 70 days. In addition, although there was no significant difference between *C. mollissima* seeds and the other three seeds in peak period, there was a certain significance in initial and end period. Therefore, there are some differences in seed rain dropping dynamics between *Castanea* and *Quercus*.

The dynamic change of seed rain is mainly affected by its own biological characteristics and local environmental factors [36,37,38]. In our study, there should be some biological differences between the *C. mollissima* of *Castanea* and the other three species of *Quercus*. For example, *C. mollissima* seeds have a dormant period and can germinate only after the dormant stage, while the other three seeds are easy to germinate and will germinate under appropriate conditions; this may reflect the adaptation strategies of different plants to the environment. Therefore, the seed rain dropping of dominant species, especially its peak, is not consistent and not completely concentrated in the same time period, which can promote the coexistence of dominant species to a large extent, ease the competition between species, and lay a foundation for maintaining forest species diversity, which is also a reproductive or adaptation strategy of plants to the environment [35,36,37]. In addition, in our study site, the seeds of the four Fagaceae species are important food sources for seed predators (especially forest rodents). Compared with the other three *Quercus* species, *C. mollissima* seeds have high nutrient and low tannin content [26,27] and are preferred by forest rodents. In this study, we found that the seed rain initial period of *C. mollissima* was later than that of the other three *Quercus* species, but the peak period was earlier than them, which may be a strategy to adapt with the competition. The lack of concentrated seed rain dropping of the four species may greatly reduce the risk of predation of large numbers of seeds [36,38], thereby promoting their own dispersal and regeneration.

### 4.2. Response of Seed Rain Dynamics to Climate Variation

Seasonal changes of climate factors, such as temperature and precipitation and sunshine length, will lead to seasonal changes in plant reproductive phenology [39]. Many studies have shown that climate factors may seriously affect plant phenology, and temperature is one of the most important factors [40,41,42,43]. The spring phenology of trees was mainly controlled by the temperature of the previous period. Usually, the higher the temperature in a certain spring, the earlier the phenology of plants; the lower temperature in spring, the later the plant phenology [7]. In addition, some research results also showed that the autumn phenology of trees was also affected by the previous temperature: usually the higher the temperature in autumn, the later the phenology of plants, and the lower the temperature in autumn, the earlier the phenology of plants [44,45,46,47]. Therefore, plant phenology is closely related to temperature, especially in the early stages of plant growth and development, and there is a significant correlation between the start date of various phenological periods and the early temperature [41,47]. In this study, the seed rain dynamics of the four plant species are closely related to temperature. For the seed rain initial period, the initial period of seed rain of *C. mollissima*, *Q. aliena,* and *Q. variabilis* all had non-linear correlation with t-2 average minimum temperature and spring season average minimum temperature, respectively, and *Q. serrata* had a non-linear positive correlation with t-3 average maximum temperature. The peak period of *C. mollissima*, *Q. aliena,* and *Q. serrata* all had non-linear correlation with t-1 average maximum temperature and t-3 average minimum temperature, respectively, and a nonlinear negative relationship between peak period and t-1 average maximum temperature. The end period of the four species all had nonlinear relationship with t-2 average maximum temperature. The above results show that the three important periods of seed rain dropping of the four plant species mainly have a nonlinear relationship with t-1, t-2, and t-3 temperature, which indicates that the temperature in the last three months of seed dropping has an important effect on seed growth and seed dropping. Too high or too low temperature may affect the absorption and accumulation of plant seeds to nutrients, and the adaptive temperature is conducive to the absorption of nutrients and seed growth. Furthermore, the seed rain dynamics of the four plants species responded differently to temperature at different time scales, which further shows that seed setting of different plants is sensitive to temperature at different time scales, and in turn, it may affect seed rain dropping.

In addition to temperature, precipitation is another important climatic factor affecting plant phenology [8,46,47,48,49,50,51]. In this study, for the initial period of *C. mollissima* and *Q. aliena*, *Q. serrata* all had a non-linear correlation with t-1 accumulated precipitation, and t-2 accumulated precipitation, respectively, and was a positive linear relation between *Q. variabilis* and t-3 accumulated precipitation. For peak period, *C. mollissima* and *Q. variabilis* had a non-linear correlation with spring season accumulated precipitation and t-1 accumulated precipitation, and *Q. aliena* had a negative linear relation between peak period and t-2 accumulated precipitation, but *Q. serrata* had a positive linear relation between peak period and t-3 accumulated precipitation. For the end period of *C. mollissima*, *Q. aliena*, *Q. variabilis,* and *Q. serrata,* they all had a non-linear correlation with t-2 accumulated precipitation autumn season accumulated precipitation and t-2 accumulated precipitation spring season accumulated precipitation, respectively. The above results show that most of the four seeds had a non-linear relationship with precipitation, indicating that appropriate precipitation is beneficial to the accumulation of plant nutrients and seed maturation, while too much or too little precipitation may affect the photosynthesis of plants, which is not conducive to seed maturation and may lead to early or delayed seed dropping.

In conclusion, the seed rain dropping dynamics of the four plant species have different sensitivity to temperature and precipitation at different time scales, which may also be the adaptation strategies of different plants species to the environment [36].

### 4.3. Symmetrically Coexisting Distribution of Different Plant Species

For plant species in their phenological period (especially the flowering period and fruiting period), the greater the difference, the smaller the niche overlap, so they can effectively avoid the competition for resources (such as pollinators and communicators fruit) and achieve the population’s fitness so as to realize species coexistence in the time and space niche [52,53,54,55]. In this study, there were some significant differences in the initial, peak, and end periods of seed rain between *C. mollissima* and the three species of *Quercus*. These results indicated that there was no flowering and fruiting concentration between *Castanea* and *Quercus*. On the one hand, it may be because there are some biological differences between *C. mollissima* of *Castanea* and the three species of *Quercus*; thus, they could well avoid fierce competition for similar resources and coexist in the same region; this may also be a reproductive strategy for plants. On the other hand, the four plants species have different sensitivity and responses to temperature and precipitation at different time scales, which indicated they can avoid fierce competition for the same environment resources. It also can facilitate staggered seed dropping, which may be conducive to better sympatric coexistence and natural regeneration of the four species. Finally, and although there were no significant differences in seed rain dynamics among the three species of *Quercus*, there are still some differences in seed rain dropping dynamics between them among the 10 years. Thus, seeds of the four speices did not drop centrally, which can reduce the risk for each individual species of being eaten by a large number of seed predators. This would be beneficial for promoting seeds dispersal, natural regeneration, and seedling establishment and enabling their successful co-existence in the same domain.

## 5. Conclusions

In this study, we monitored seed rain dynamics of four sympatrically distributed Fagaceae species in the Qinling Mountains of China for 10 consecutive years and also analyzed the effect of climate factors on seed rain dynamics. We found there were significant differences in the seed rain dynamics especially between *C. mollissima* of *Castanea* and the other three species of *Quercus* in the initial period and end period and the duration of the whole seed rain process among the ten years. This indicated that there were certain differences in biological characteristics between them, and there was no concentrated flowering and fruiting among plants of different genera. Consequently, they could well avoid fierce competition for similar resources and coexist in the same region. This may also be a reproductive strategy for plants. In addition, we found the seed rain dropping dynamics of the four species had different sensitivity and response to temperature and precipitation, which indicated that they can avoid fierce competition for the same environmental resources. Moreover, the seeds of the four plants were important food sources for forest rodents within the study site. This may also be a reproductive strategy for the species, as the differences of seed rain dropping dynamics could reduce the risk of being eaten in large numbers by seed predators, thereby promoting their own dispersal and regeneration. All of the above findings contribute to their better coexistence in the same domain.

## Data Availability

The climatic variables from Foping on the south slopes of the Qinling Mountains in Shaanxi Province, central China, during 2011–2020, including temperature and precipitation, were derived from the China Meteorological Data Service Center (CMDC) (http://data.cma.cn/) (accessed on 1 March 2021), which is publicly accessible.
